# The MRN protein complex genes: *MRE11* and *RAD50* and susceptibility to head and neck cancers

**DOI:** 10.1186/1476-4598-12-113

**Published:** 2013-09-30

**Authors:** Iwona Ziółkowska-Suchanek, Maria Mosor, Małgorzata Wierzbicka, Małgorzata Rydzanicz, Marta Baranowska, Jerzy Nowak

**Affiliations:** 1 Department of Molecular Pathology, Institute of Human Genetics, Polish Academy of Sciences, Strzeszyńska 32 St, 60-479 Poznań, Poland; 2 Department of Otolaryngology and Laryngeal Oncology, K. Marcinkowski University of Medical Sciences, Przybyszewskiego 49 St, 60-355, Poznań, Poland

**Keywords:** DNA repair genes, Cancer susceptibility, Laryngeal cancer, Multiple primary tumors of head and neck

## Abstract

**Background:**

The members of MRE11/RAD50/NBN (MRN) protein complex participates in DNA double-strand break repair and DNA-damage checkpoint activation. We have previously shown that the p.I171V *NBN* gene mutation may contribute to the development of laryngeal cancer. This study tested the hypothesis that variants of the *MRE11* and *RAD50* genes, previously described as cancer risk factors, predispose to increased susceptibility to head and neck cancer.

**Findings:**

In this study we analyzed the *RAD50* and *MRE11* genes in 358 patients: 175 with a single laryngeal cancer (LC), 115 with multiple primary tumors but one malignancy (primary or second) localized in the larynx (MPT-LC), 68 patients with multiple primary tumors localized in the head or neck (MPT) and 506 controls. No carriers of previously reported mutation in the *MRE11* or *RAD50* gene (particularly the pathogenic c.687delT) were detected in the present study. We identified the p.V127I variant (2/175 LC, 2/506 controls; OR=2.91; 95% CI 0.41-20.85) and p.V315L variant (2/115 MPT-LC, 1/506 controls; OR=8.96; 95% CI 0.81-99.68) of the *RAD50* gene.

**Conclusions:**

Our data indicated that previously described common genetic variations in the *MRE11* and *RAD50* genes do not contribute to an increased risk of laryngeal cancer and second primary tumors localized in the head and neck. Prospective studies with larger groups of patients may reveal the possible impact of these genes in tumor occurrence.

## Findings

### Introduction

Head and neck squamous cell carcinoma (HNSCC) is the sixth most common cancer worldwide and represent approximately 3% of all malignancies [1]. Despite improvements in the diagnosis and treatment of HNSCC, survival rates of patients with these tumors still remain at low levels (40-50%), mainly because of the occurrence of second primary tumors (SPT) [2]. In the etiology of head and neck cancers and SPT smoking and alcohol abuse (“condemned mucosa theory”) are essential but not the only causal factors. Vast majority of patients are current/former smoker and are exposed to many mutagenic agents which can induce DNA double-strand breaks (DSB). The MRN complex is involved in DSB repair by homologous recombination or non-homologous end joining (NHEJ), telomere maintenance, meiotic recombination, and DNA damage response [3]. Heterozygous mutations carriers in genes of the MRN complex seem to be predisposed to cancer development, especially heterozygous *NBN* mutation carriers have an elevated risk of acute lymphoblastic leukemia [4], melanoma, colon and rectum cancer, prostate and breast cancer [5]. The c.687delT *RAD50* mutation was reported with significantly elevated frequency in breast cancer patients from Finland [5-7]. Molecular variants of the *MRE11* gene have been identified in breast and ovarian cancer [5,6,8]. We have previously shown that the heterozygous p.I171V mutation of the *NBN* gene may contribute to laryngeal cancer (OR=11.7, 95% CI 1.3–105.2) and multiple primary tumors (OR=28.35, 95% CI 3.27–245.7) [9]. Likewise, in our next studies we found that specific haplotypes of the *NBN* gene may be associated with the same cancer patients [10]. These data prompted us to evaluate the possible role of the two rest subunits of the MRN protein complex, the *MRE11* and *RAD50* genes, in head and neck cancer susceptibility, especially there are no reports related to this topic.

### Patients and controls

Blood samples were collected from 358 Polish patients: 175 with a single laryngeal cancer (LC) and 115 with multiple primary tumors but one malignancy (primary or second primary) localized in the larynx (MPT-LC) and 68 multiple primary tumors localized in the head or neck (MPT). The particular characteristics of MPT-LC/MPT patients were described in our previous studies [9,10]. None of 175 LC patients with a single laryngeal cancer developed any second primary tumor during 5 years of observation. All patients included in the MPT-LC/MPT group fulfilled all the criteria proposed by Warren and Gates [2] and accepted by the International Agency for Research on Cancer (IARC). In all 115 MPT-LC patients, either the index or the second tumor was laryngeal cancer. In total, 506 anonymous blood samples collected on Guthrie cards, matched regionally to cancer patients were used as population controls. All patients signed informed consent forms approved by the Ethics Committee of the University of Medical Sciences in Poznań.

## Methods

The PCR-MSSCP method was used to analyze the coding sequence and exon-intron boundaries of exon 3, 4, 5, 7, 21 and 25 of the *RAD50* gene as described in our previous study [11] and exon 5, 8, 9, 10, 14, 15, 17 and 19 of the *MRE11* gene. The selection of the screened regions was based on the reported occurrence of the mutations in cancer in former studies. All samples were analyzed by multitemperature single-strand conformation polymorphism (MSSCP) method (Biovectis, Warsaw, Poland). Samples that showed an aberrant shift in MSSCP were sequenced (OLIGO, IBB, Warszawa). The significance of differences between studied groups was assessed by the *χ*^2^ test or Fischer’s exact test, depending on variants’ frequency (GraphPad Software Inc. San Diego, CA). Crude odds ratios (ORs) were calculated and given with 95% confidence intervals (CIs). The differences were considered significant if the value of probability (*P*) less than 0.05. To account for false-positive findings, multiple testing correction was carried out by Bonferroni correction [*P*-value * n (number of genes in test) <0.05] [12]. Because we tested 2 genes, the gene will be significant if the corrected *P*-value is below the cutoff of <0.025.

## Results

The *RAD50* c.687delT was not observed among 358 cancer patients and 506 controls. We have identified 3 heterozygous sequence variants (Table 1). Two of them were missense variants (p.V127I and p.V315L). These two missense variants were evaluated for possible functional effect on RAD50 protein by SIFT and PolyPhen analysis [11] which suggested that both variants are predicted to be tolerated. The c.3876C>T variant was synonymous (p.N1292N). No significant differences in variants frequencies were observed when comparing 2 groups of cancer patients with controls (Table 1). In intron 4 we have identified one polymorphism c.551+19G>A (rs17166050) (Table 2), in which heterozygous genotype GA was significantly more frequent, even after multiple testing correction, in controls than in MPT group (*P*=0.0205). We did not find any sequence variants in the *MRE11* gene, beside intronic c.1783-86delAG (Table 1).

**Table 1:** **The *RAD50 *and *MRE11 *gene variants detected in laryngeal cancer (LC), multiple primary tumors with laryngeal cancer (MPT-LC), multiple primary tumors of head and neck (MPT) and controls**

**Gene**	**Exon/intron**	**Nucleotide change**	**Aminoacid change**	**LC** **n = 175** **OR (95% CI)**	**MPT-LC** **n = 115** **OR (95% CI)**	**MPT** **n = 68** **OR (95% CI)**	**Controls** **n = 506**
*RAD50*	Ex. 4	c.379G>A	p.V127I	2	-	-	2
				2.91 (0.41-20.85)			
	Ex. 7	c.943G>T	p.V315L	-	2	-	1
					8.96 (0.81-99.68)		
	Ex. 25	c.3876C>T	-	1	-	-	0/180
				8.71 (0.35-215)			
*MRE11*	Int. 16	c.1783-86delAG	-	39	18	20	61/279
				1.03 (0.65-1.62)	0.66 (0.37-1.18)	1.49 (0.82-2.69)	

**Table 2:** **The *RAD50 *_rs17166050 genotypes/allele frequency distribution and logistic regression analysis (with odds ratio and 95% confidence interval) in laryngeal cancer (LC), multiple primary tumors with laryngeal cancer (MPT-LC), multiple primary tumors of head and neck (MPT) and controls**

**Genotype/allele**	**Controls** **n (%)**	**LC** **n (%)**	**OR (95% CI)**	**MPT-LC** **n (%)**	**OR (95% CI)**	**MPT** **n (%)**	**OR (95% CI)**
GG	233 (47)	87 (50)	1^*^	50 (44)	1^*^	24 (35)	1^*^
GA	195 (40)	69 (39)	1.06 (0.74-1.53)	57 (50)	0.73 (0.48-1.12)	38 (56)	**0.53 (0.31-0.92)**^**†**^
AA	66 (13)	19 (11)	1.30 (0.74-2.29)	8 (6)	1.78 (0.80-3.92)	6 (9)	1.13 (0.44-2.89)
GA+AA	261 (52)	88 (50)	1.11 (0.78-1.56)	65 (57)	0.86 (0.57-1.30)	44 (65)	0.61 (0.36-1.04)
G	661 (66)	243 (69)	1^*^	157 (68)	1^*^	86 (63)	1^*^ 0.85
A	327 (34)	107 (31)	1.12 (0.87-1.46)	73 (22)	1.06 (0.78-1.45)	50 (37)	(0.59-1.24)

^*^Reference category; OR (95% CI) – odds ratio (95% confidence interval); ^†^ Result statistically significant (*p*=0.0205).

## Discussion

In the current preliminary study we screened the selected regions, where most of already known molecular variants of the *RAD50* and *MRE11* gene occur, among 358 head and neck cancer patients and 506 controls. To our knowledge the first prospective investigation of its kind, we have shown that *MRE11* and *RAD50* genes do not contribute to laryngeal cancer. The c.687delT *RAD50* mutation, not identified in our study, was reported with significantly elevated frequency in breast cancer patients from Finland [5-7]. This pathogenic mutation generates a truncated protein without the important C-terminal site and it is a founder mutation in Finland, which increases 4-fold risk of breast cancer in Finnish women [7]. However, the occurrence of the c.687delT mutation in other populations has been difficult to confirm among non-BRCA1/2 hereditary breast cancer patients from the UK [13] and France [14]. Similarly the deletion was not detected in our previous study among Polish non-selected breast cancer patients [11], which is in agreement with results of the current study. It seems that pathogenic variants of the *RAD50* gene may have a impact to cancer development only in certain populations, like Finnish.

The potential role of the *MRE11* gene in human cancers is not well documented. Only J. Bartkova et al. have identified two germline mutations: a missense mutation p.R202G and a truncating mutation p.R633X, which qualify the *MRE11* as a candidate breast cancer susceptibility gene in a subset of non-BRCA1/2 families in Denmark [8]. We did not find any mutations in our group of subjects which confirm that genetic variants of the *MRE11* gene among cancer patients are relatelively rare. However aberrant expression has been commonly observed and it is supposed that *MRE11* overexpression may be the mechanism increasing risk of malignancy development.

Beside mutations and pathogenic variants of the MRN genes, sets of single nucleotide polymorphisms (SNP) were tested for association with many cancers such non-Hodgkin lymphoma (NHL) [15], breast cancer [16] and bladder cancer [17]. J. Schuetz et al. find that two variants in *RAD50* were suggestive of association with specific non-Hodgkin lymphoma (NHL) European cases, but there were not significant after correction of multiple tests. In the same study, the rs17166050 polymorphism was detected in intron 4 of the *RAD50* gene and showed no association with NHL [15]. Similar results were reported in our current and previous study among non-selected breast cancer patients [11]. In our another study we confirmed the association of the variant allele of the *RAD50* rs171660505 with decreased risk of the childhood acute lymphoblastic leukemia [18]. A. Choudhury and colleagues have genotyped SNPs in DSB signalling genes and found an marginally association of the *MRE11* 3′UTR SNP rs2155209 with bladder cancer [17]. In another study, H. Hsu et. al has excluded any association of the *RAD50/MRE11* polymorphisms and breast cancer risk, beside one SNP in *NBN*[16]. However, an increased risk of developing breast cancer was found in women harboring a greater number of putative high-risk genotypes of all MRN genes. It seems that genes which are not associated with cancer independently, could modify cancer risk jointly or in combination with other variants.

In conclusion, current results demonstrate that *RAD50/MRE11* variants occur at very low frequency in analyzed group of cancer patients in Poland. It seems that only the p.I171V *NBN* gene may be associated with head and neck cancer. However, the lack of evidence of the common *RAD50/MRE11* gene variants in this preliminary study, should be verified in replication studies. Depending on genetic heterogeneity of head and neck tumors and population diversity, prospective studies with larger groups of patients may reveal the possible impact of the MRN complex genes in tumor occurrence.

## Abbreviations

MRN: MRE11/RAD50/NBN protein complex; LC: Laryngeal cancer; MPT-LC: Multiple primary tumors with laryngeal cancer; MPT: Multiple primary tumors localized in the head or neck; HNSCC: Head and neck squamous cell carcinoma; SPT: Second primary tumors; DSB: DNA double-strand breaks; NHEJ: Non-homologous end joining; MSSCP: Multitemperature single-strand conformation polymorphism; SNP: Single nucleotide polymorphism; NHL: Non-Hodgkin lymphoma.

## Competing interests

The authors have declared no conflicts of interest.

## Authors’ contributions

IZS and MM designed methods and experiments, carried out the laboratory experiments, analyzed the data and interpreted the results. IZS drafted the manuscript. MW and MR carried out patient recruitment, sample collection and suggestions for finalization of the manuscript. MB co-worked on the laboratory experiments and analyzed the data. JN participated in study coordination and manuscript preparation. All authors read and approved the manuscript.
